# Identification and fine mapping of *qGR6.2*, a novel locus controlling rice seed germination under salt stress

**DOI:** 10.1186/s12870-020-02820-7

**Published:** 2021-01-09

**Authors:** Peng Zeng, Peiwen Zhu, Luofeng Qian, Xumei Qian, Yuxin Mi, Zefeng Lin, Shinan Dong, Henrik Aronsson, Hongsheng Zhang, Jinping Cheng

**Affiliations:** 1 State Key Laboratory of Crop Genetics and Germplasm Enhancement, Jiangsu Collaborative Innovation Center for Modern Crop Production, Cyrus Tang Innovation Center for Crop Seed Industry, Nanjing Agricultural University, Nanjing, China; 2 Department of Biological and Environment Sciences, University of Gothenburg, Gothenburg, Sweden

**Keywords:** Rice, Seed germination, Salt stress, Quantitative trait loci (QTLs), Fine mapping, Marker-assisted selection (MAS)

## Abstract

**Background:**

Rice growth is frequently affected by salinity. When exposed to high salinity, rice seed germination and seedling establishment are significantly inhibited. With the promotion of direct-seeding in Asia, improving rice seed germination under salt stress is crucial for breeding.

**Results:**

In this study, an *indica* landrace Wujiaozhan (WJZ) was identified with high germinability under salt stress. A BC_1_F_2_ population derived from the crossing WJZ/Nip (*japonica*, Nipponbare)//Nip, was used to quantitative trait loci (QTL) mapping for the seed germination rate (GR) and germination index (GI) under H_2_O and 300 mM NaCl conditions. A total of 13 QTLs were identified, i.e. ten QTLs under H_2_O conditions and nine QTLs under salt conditions. Six QTLs, *qGR6.1*, *qGR8.1*, *qGR8.2*, *qGR10.1*, *qGR10.2* and *qGI10.1* were simultaneously identified under two conditions. Under salt conditions, three QTLs, *qGR6.2*, *qGR10.1* and *qGR10.2* for GR were identified at different time points during seed germination, which shared the same chromosomal region with *qGI6.2*, *qGI10.1* and *qGI10.2* for GI respectively. The *qGR6.2* accounted for more than 20% of phenotypic variation under salt stress, as the major effective QTL. Furthermore, *qGR6.2* was verified via the BC_2_F_2_ population and narrowed to a 65.9-kb region with eleven candidate genes predicted. Based on the microarray database, five candidate genes were found with high transcript abundances at the seed germination stage, of which *LOC_Os06g10650* and *LOC_Os06g10710* were differentially expressed after seed imbibition. RT-qPCR results showed the expression of *LOC_Os06g10650* was significantly up-regulated in two parents with higher levels in WJZ than Nip during seed germination under salt conditions. Taken together, it suggests that *LOC_Os06g10650*, encoding tyrosine phosphatase family protein, might be the causal candidate gene for *qGR6.2*.

**Conclusions:**

In this study, we identified 13 QTLs from a landrace WJZ that confer seed germination traits under H_2_O and salt conditions. A major salt-tolerance-specific QTL *qGR6.2* was fine mapped to a 65.9-kb region. Our results provide information on the genetic basis of improving rice seed germination under salt stress by marker-assisted selection (MAS).

**Supplementary Information:**

The online version contains supplementary material available at 10.1186/s12870-020-02820-7.

## Background

Soil salinity is the primary abiotic stress affecting crop growth and productivity worldwide [1]. It is estimated that 6% of the Earth’s landmass and 20% of irrigated land are affected by salinity [2]. Rice is the most important staple food, feeding more than half of the world’s population. Compared to wheat, rice is more sensitive to salt stress, and approximately 30% of the rice-growing area in the world is affected by salinity [3]. According to previous reports, high salinity inhibits seed germination and seedling establishment, reduces plant growth and diminishes rice yield [4, 5]. Although saline soil could be improved by large-scale irrigation, drainage schemes, and chemical treatment, all these solutions are overly costly [6]. Hence, genetic improvement of salt tolerance has been an important and feasible objective for rice breeding in coastal areas [7].

Salt tolerance is a polygenic trait and highly influenced by the environment [8–10]. To date, hundreds of salt-response QTLs have been reported at different developmental growth stages in rice [11]. Lin et al. [12] detected two major QTLs (*qSKC-1* and *qSNC-7*) for Na^+^/K^+^ content in the seedling shoot on chromosomes 1 and 7. Based on the results of QTL mapping, the major QTL *qSKC-1* has been cloned, which encodes an HKT-type transporter protein regulating K^+^ content in the shoot [13]. Another major QTL, *Saltol*, was identified and used to breed new salt-tolerant varieties by MAS, such as Pusa44, CR1009, and PR114, which enhances salt tolerance at the seedling stage [14, 15].

Salt tolerance at the seed germination stage is not consistently related to other stages, such as the seedling and reproductive stages [9, 16]. Seed germination is a key parameter of prime significance, and fundamental to total biomass and yield in a plant’s life cycle, starting with the uptake of water followed by the protrusion of the radicle through the seed envelopes [17]. During seed germination, salinity results in many disorders and metabolic changes such as changed the enzymes activity causing high solute leakage [18], decreased K^+^ efflux and impeding α-amylase activity [19]. Few studies have aimed at genetically dissecting seed germination under salt stress in rice. Wang et al. [4] detected 16 QTLs of rice seed germination ability at 100 mM NaCl from the *japonica* variety Jiucaiqing. Approximately 50 loci have been identified for seed germination under salt stress by genome-wide association studies (GWAS) [20–22]. Fujino et al. [23] reported that *qLTG3–1* was associated with germination under low temperature through tissue vacuolation and weakening, and also with good seed germinability under high salinity. Recently, a QTL *qSE3* promoting seed germination and seedling establishment was identified from a *japonica* landrace Jiucaiqing at 300 mM NaCl, which encodes a potassium transport *OsHAK21* and mediates seed germination under salt stress through abscisic acid (ABA) metabolism [24]. With the increasing promotion of rice direct-seeding methods, it is of considerable importance to explore more loci or genes for seed germination under salt stress and develop cultivars with a high capacity for seed germination under salt stress by MAS in coastal areas.

In this study, an *indica* landrace WJZ from Yunan province in China [21] was identified with a high capacity for seed germination under high salt stress. To identify QTL for seed germination, the germination rate (GR) and germination index (GI) of the BC_1_F_2_ population derived from a cross between WJZ and Nip were evaluated under H_2_O and 300 mM NaCl conditions. A major QTL *qGR6.2* on the short arm of chromosome 6 was specifically identified under 300 mM NaCl conditions. Additionally, *qGR6.2* was verified among the BC_2_F_2_ population and fine mapped within a 65.9-kb region between Z654 and Z619 markers. This work could be valuable in elucidating the genetic and molecular basis of seed germination under salt stress.

## Results

### Characteristics of seed germination for two parents under salt stress

The germination rate (GR), seedling percentage (SP) and germination index (GI) for *indica* WJZ and *japonica* Nip seeds were evaluated after 10 days (d) of imbibition under H_2_O and various salt concentration conditions (150, 200, 250, 300 and 350 mM NaCl). Both WJZ and Nip germinated readily, with approximately 100% of the GR and SP for WJZ and Nip under H_2_O conditions (Table 1). However, WJZ had a significantly higher GI (13.93) than Nip (10.51), indicating that WJZ germinated faster than Nip under H_2_O conditions. Under various NaCl concentrations, there was a significant decrease in GR, SP, or GI of both WJZ and Nip (Table 1), indicating that rice seed germination was inhibited and delayed by salt stress. When exposed to 350 mM NaCl, WJZ seeds displayed 80.03% of GR, in contrast to 12.22% of GR for Nip (Table 1), suggesting that WJZ was considerably more salt-tolerant than Nip during seed germination. Considering the greatest variation in GR, SP and GI between the two rice parents, seed germination under salt stress was assessed with 300 mM NaCl in later experiments.

**Table 1:** Phenotypic values of seed germination for the two parents under different NaCl concentration conditions

NaCl concentration (mM)	GR (%)		SP (%)		GI	
	WJZ	Nip	WJZ	Nip	WJZ	Nip
0	100.00±0.00	99.33±0.82	100.00±0.00	99.33±0.82	13.93±0.43^b^	10.51±0.44
150	99.33±0.82^b^	87.77±2.74	99.33±0.82^a^	85.57±3.60	12.00±0.19^b^	6.62±0.30
200	98.67±0.82^a^	86.63±4.08	96.67±2.37^b^	68.90±1.35	10.47±0.11^b^	4.96±0.13
250	95.53±2.74^a^	77.77±4.90	88.90±7.20^b^	51.13±3.60	8.24±0.79^b^	3.97±0.11
300	95.53±2.74^b^	34.43±3.60	65.53±7.58^b^	1.11±0.82	6.92±0.25^b^	1.79±0.10
350	80.03±10.80^b^	12.22±3.61	26.67±8.17^a^	0.00±0.00	5.09±0.71^b^	0.76±0.15

*GR* germination rate, *SP* seedling percentage, *GI* germination index, *WJZ* Wujiaozhan, *Nip* Nipponbare

^a^ and ^b^ indicate significant differences compared to WJZ at the 5 and 1% levels, respectively

**Fig. 1:**
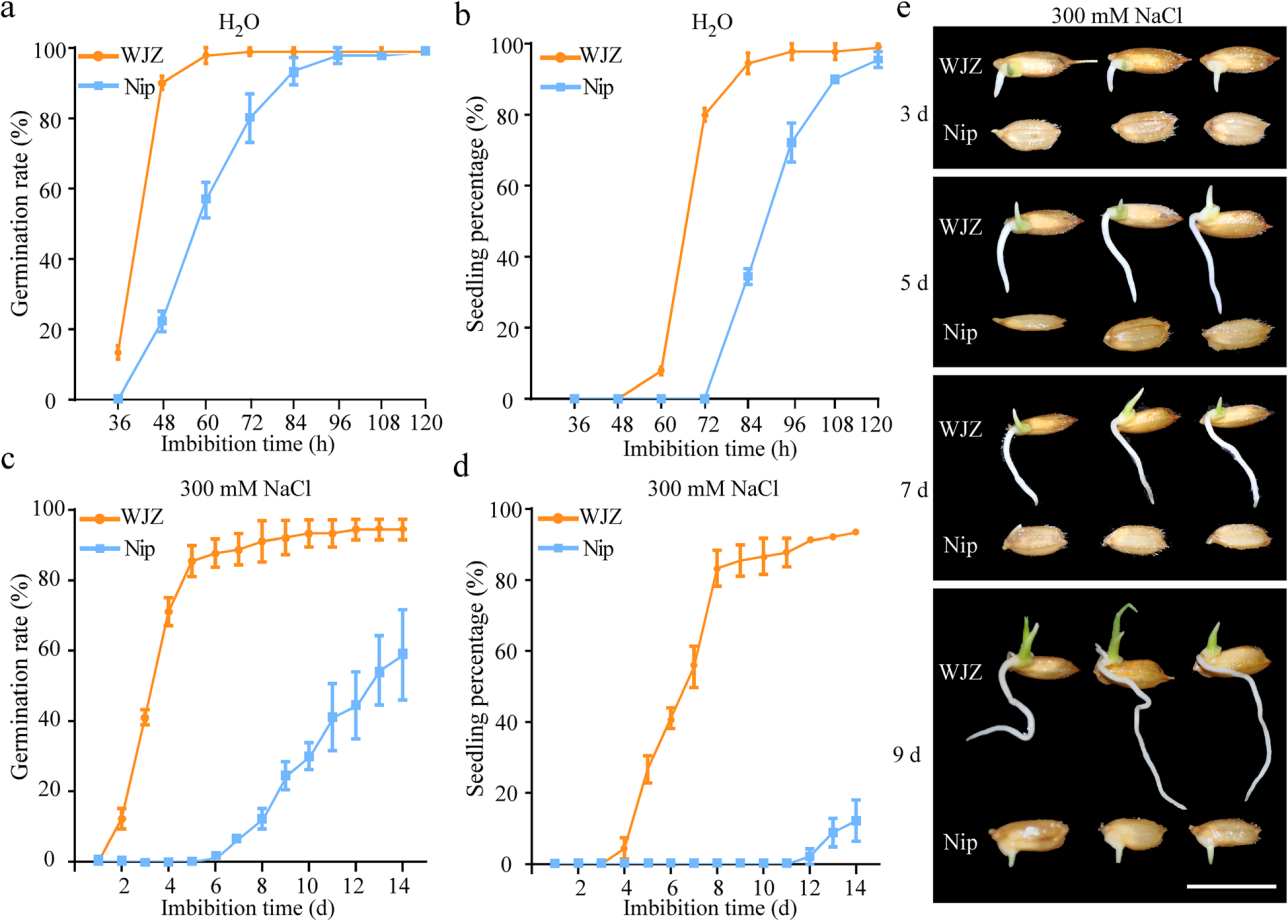
Evaluation of seed germination and seedling establishment between WJZ and Nip under H_2_O and 300 mM NaCl conditions. **a**-**b** Quantification and statistical analysis of germination rate (**a**) and seedling percentage (**b**) of two-parent varieties from 36 h to 120 h under H_2_O conditions. **c**-**d** Quantification of germination rate (**c**) and seedling percentage (**d**) of two-parent varieties from 1 to 14 d under 300 mM NaCl conditions. Each point represents the mean ± standard deviation. **e** Morphology of two varieties during seed germination after 3, 5, 7 and 9 d imbibition under 300 mM NaCl conditions. Scale bar = 1 cm

To better understand the characteristics of the high seed germination ability and salt tolerance of WJZ, the differences in GR and SP between the two parents were further analyzed under H_2_O and 300 mM NaCl conditions. Under H_2_O conditions, although all seeds of both parents germinated and established seedlings after 120 h (5 d) of imbibition (Fig. 1a, b), WJZ germinated faster and had higher values of GR and SP than Nip at the beginning of seed germination. Under 300 mM NaCl conditions, significant differences in GR and SP between WJZ and Nip were observed from 3 to 14 d during seed germination (Fig. 1c-e). The WJZ began to germinate after 2 d of imbibition, and its GR reached 90% after 7 d of imbibition (Fig. 1c), with a strong seedling establishment capacity being observed (Fig. 1d, e). However, Nip started to germinate after 7 d of imbibition and showed only 58.89% GR after 14 d of imbibition (Fig. 1c).

### Variation in seed germination traits among the BC_1_F_2_ populations

**Fig. 2:**
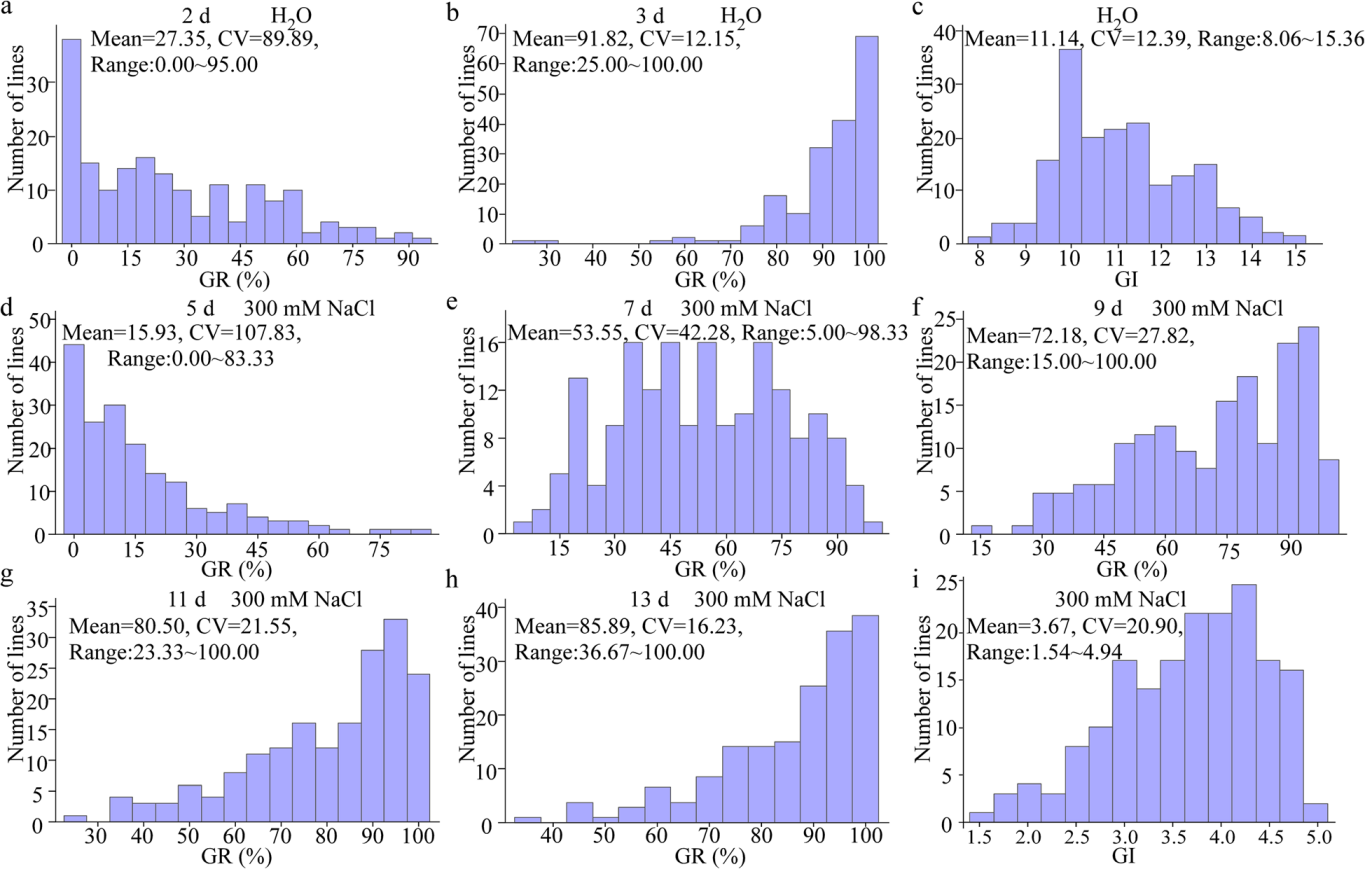
Performance of seed germination traits in the BC_1_F_2_ population on different days under H_2_O and 300 mM NaCl conditions. Performance of GR at 2 d (**a**) and 3 d (**b**) imbibition, and GI (**c**) under H_2_O conditions, GR at 5 d (**d**), 7 d (**e**), 9 d (**f**), 11 d (**g**), 13 d (**h**) imbibition and GI (**i**) under 300 mM NaCl conditions. The mean, range and CV (coefficient of variation) of GR and GI were listed in figure

A BC_1_F_2_ population consisting of 181 individuals was derived from the crossing WJZ/Nip (*japonica*, Nipponbare)//Nip (Fig. S1a). The variations in GR and GI among this BC_1_F_2_ population under H_2_O and 300 mM NaCl conditions were analyzed. All the traits observed, including GR at 2 d, 3 d and GI under H_2_O conditions, GR at 5 d, 7 d, 9 d, 11 d, 13 d and GI under 300 mM NaCl conditions, showed a continuous distribution and had a wide range of genetic variations (Fig. 2). Under H_2_O conditions, there was a left-skewed distribution of GR at 2 d, a right-skewed distribution at 3 d (Fig. 2a, b), and a symmetrical distribution of GI (Fig. 2c). Under 300 mM NaCl conditions, GR showed a left-skewed distribution at 5 d, a right-skewed distribution at 9, 11 and 13 d during seed germination (Fig. 2d, f-h), and a symmetrical distribution at 7 d (Fig. 2e). The GI under 300 mM NaCl conditions showed a right-skewed distribution (Fig. 2i). These results indicated that the traits of GR and GI are polygenic characteristics and might be regulated by various genes at the early and later stages of germination under H_2_O or NaCl conditions.

### QTL mapping of seed germination traits under H_2_O and salt conditions

A molecular linkage map was constructed with the above BC_1_F_2_ population for QTL mapping of seed germination traits (GR and GI) under H_2_O and 300 mM NaCl conditions. Under H_2_O conditions, eight QTLs for GR were identified on chromosomes 3, 6, 8 and 10, and two QTLs for GI were identified on chromosomes 6 and 10 (Table 2). GR at 2 d was associated with three QTLs (*qGR8.1*, *qGR8.2* and *qGR10.1*), and GR at 3 d was associated with six QTLs (*qGR3.1*, *qGR3.2*, *qGR3.3*, *qGR6.1*, *qGR10.1* and *qGR10.2*). The phenotypic variation explained (PVE) of GR by a single QTL ranged from 7.32 to 23.99%. One major QTL, *qGR6.1* for GR accounted for 23.99% of phenotypic variation. The *qGI6.1* and *qGI10.1* for the GI accounted for 10.39 and 8.86% of phenotypic variation, respectively. By comparison, *qGR6.1* and *qGI6.1* shared the same interval of RM190~Z602 on chromosome 6, and *qGR10.1* and *qGI0.1* shared the same interval of W13~W20 on chromosome 10 (Table 2). The additive effects of all these QTLs detected under H_2_O conditions were negative, ranging from − 0.36 to − 9.95 (Table 2), suggesting that the positive alleles were derived from WJZ.

**Table 2:** QTLs analysis of seed germination traits among BC_1_F_2_ population under H_2_O and 300 mM NaCl conditions

Treatments	Traits	QTLs	Days after imbibition	Chr.	Left Marker	Right Marker	LOD	PVE (%)	Add	Dom
H_2_O (control)	GR	*qGR3.1*	3	3	Y25	W33	3.22	7.32	−4.92	5.87
		*qGR3.2*	3	3	RM6832	RM3513	4.00	9.36	−5.52	6.08
		*qGR3.3*	3	3	RM3513	RM8277	3.57	9.75	−5.35	4.77
		*qGR6.1*	3	6	RM190	Z602	6.11	23.99	−8.51	3.73
		*qGR8.1*	2	8	RM3572	RM6208	3.32	15.75	−6.69	−14.58
		*qGR8.2*	2	8	RM6208	Y61	3.42	14.97	−8.81	−11.51
		*qGR10.1*	2	10	W13	W20	3.33	9.25	−9.95	−1.11
			3	10	W13	W20	3.33	8.95	−3.93	2.89
		*qGR10.2*	3	10	W20	RM6824	3.36	8.66	−4.01	3.10
	GI	*qGI6.1*		6	RM190	Z602	3.69	10.39	−0.44	0.04
		*qGI10.1*		10	W13	W20	3.28	8.86	−0.36	0.06
300 mM NaCl	GR	*qGR6.1*	13	6	RM190	Z602	3.14	7.07	−5.40	4.54
		*qGR6.2*	7	6	Z604	RM276	8.80	20.14	−1.86	−19.02
			9	6	Z604	RM276	10.56	23.82	−1.30	−18.69
			11	6	Z604	RM276	9.93	22.18	−1.24	−15.48
			13	6	Z604	RM276	10.58	23.54	−1.19	−12.69
		*qGR8.1*	5	8	RM3572	RM6208	4.00	18.80	−7.76	−8.00
		*qGR8.2*	5	8	RM6208	Y61	3.84	14.48	−7.17	−6.24
		*qGR10.1*	5	10	W13	W20	3.89	11.74	−7.65	−3.37
			7	10	W13	W20	5.69	14.07	−11.00	2.90
			9	10	W13	W20	6.19	14.90	−9.81	3.75
			11	10	W13	W20	6.59	16.22	−8.63	4.33
			13	10	W13	W20	5.94	14.35	−6.54	3.15
		*qGR10.2*	7	10	W20	RM6824	4.40	9.49	−9.66	0.59
			9	10	W20	RM6824	5.35	11.20	−9.30	0.92
			11	10	W20	RM6824	5.78	12.16	−8.36	1.08
			13	10	W20	RM6824	5.53	11.52	−6.52	1.19
		*qGI6.2*		6	Z604	RM276	11.20	24.39	−0.05	−0.72
	GI	*qGI10.1*		10	W13	W20	7.50	17.41	−0.41	0.14
		*qGI10.2*		10	W20	RM6824	6.50	13.18	−0.38	0.05

*GR* germination rate, *GI* germination index, *Chr.* chromosome, *LOD* the likelihood of odds, *PVE* phenotypic variation explained by each QTL, *ADD* additive effect is the effect of substituting a WJZ allele for a Nip allele, and its negative value indicates that WJZ contains the positive allele, *DOM* dominance effect

**Fig. 3:**
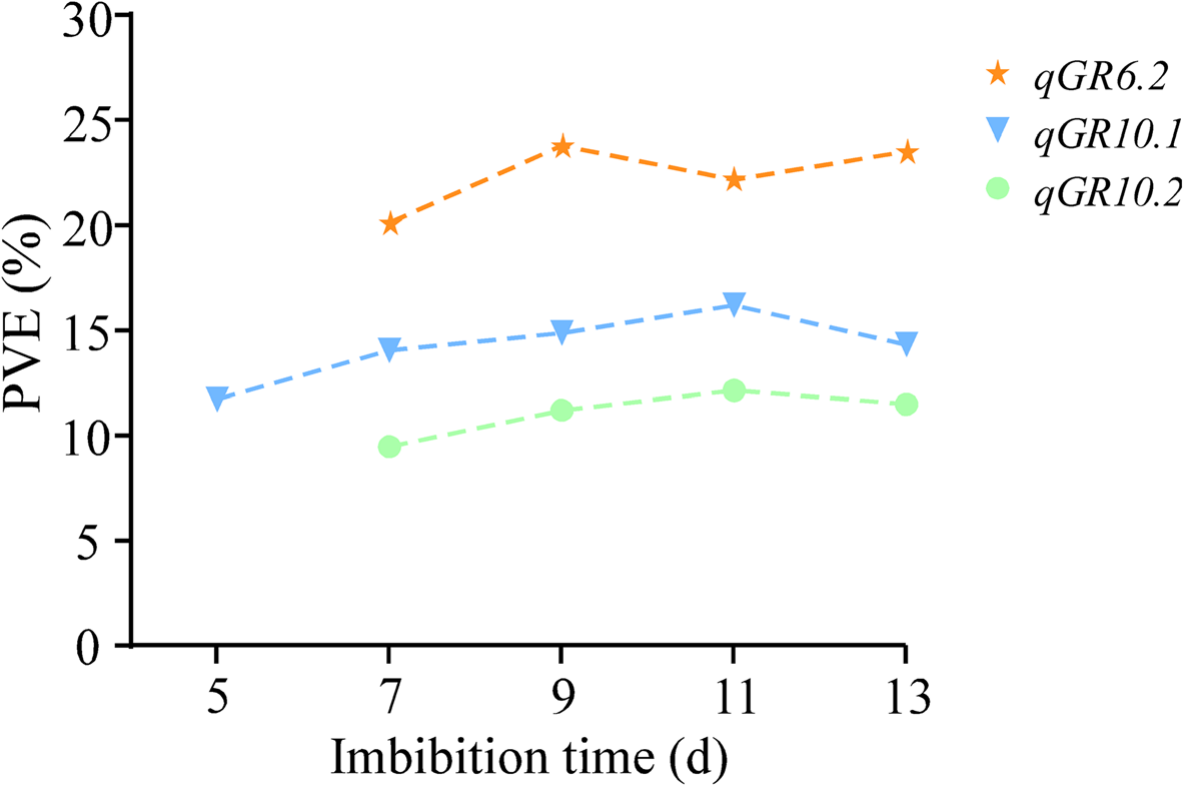
QTLs associated with GR in the BC_1_F_2_ population under 300 mM NaCl conditions from 5 to 13 d. PVE, phenotypic variation explained by QTL. Dashed lines show the trends of PVE for GR

Under 300 mM NaCl conditions, six QTLs for GR and three for GI of seed germination were identified on chromosomes 6, 8, and 10, respectively (Table 2). All these QTLs showed a negative additive effect, indicating that the positive alleles originated from WJZ. Among the six QTLs for GR, *qGR6.2* and *qGR10.2* were continuously identified at 7, 9, 11, and 13 d of seed imbibition, *qGR10.1* at 5, 7, 9, 11 and 13 d (Fig. 3), *qGR8.1* and *qGR8.2* only at 5 d, and *qGR6.1* only at 13 d. It suggested that *qGR6.2*, *qGR10.1* and *qGR10.2* might be key QTLs of seed germination under salt stress (Table 2). A major-effective QTL *qGR6.2* was flanked by Z604 and RM276, accounting for more than 20.0% of phenotypic variation. Three QTLs for GI, *qGI6.2*, *qGI10.1* and *qGI10.2*, accounted for 24.39, 17.41 and 13.18% of phenotypic variation, respectively (Table 2). By comparison, *qGR6.2* was co-localized with *qGI6.2* between Z604 and RM276 on chromosome 6, and *qGR10.1* shared the same region with *qGI10.1* in the interval of W13~W20 on chromosome 10, *qGR10.2* was co-localized with *qGI10.2* between W20 and RM6824 on chromosome 10. Among those QTLs, the major QTL *qGR6.2* or *qGI6.2* with a high LOD value (> 8) could specifically enhance GR or GI of seeds under salt conditions (Table 2).

### Validation and fine mapping of *qGR6.2*

**Fig. 4:**
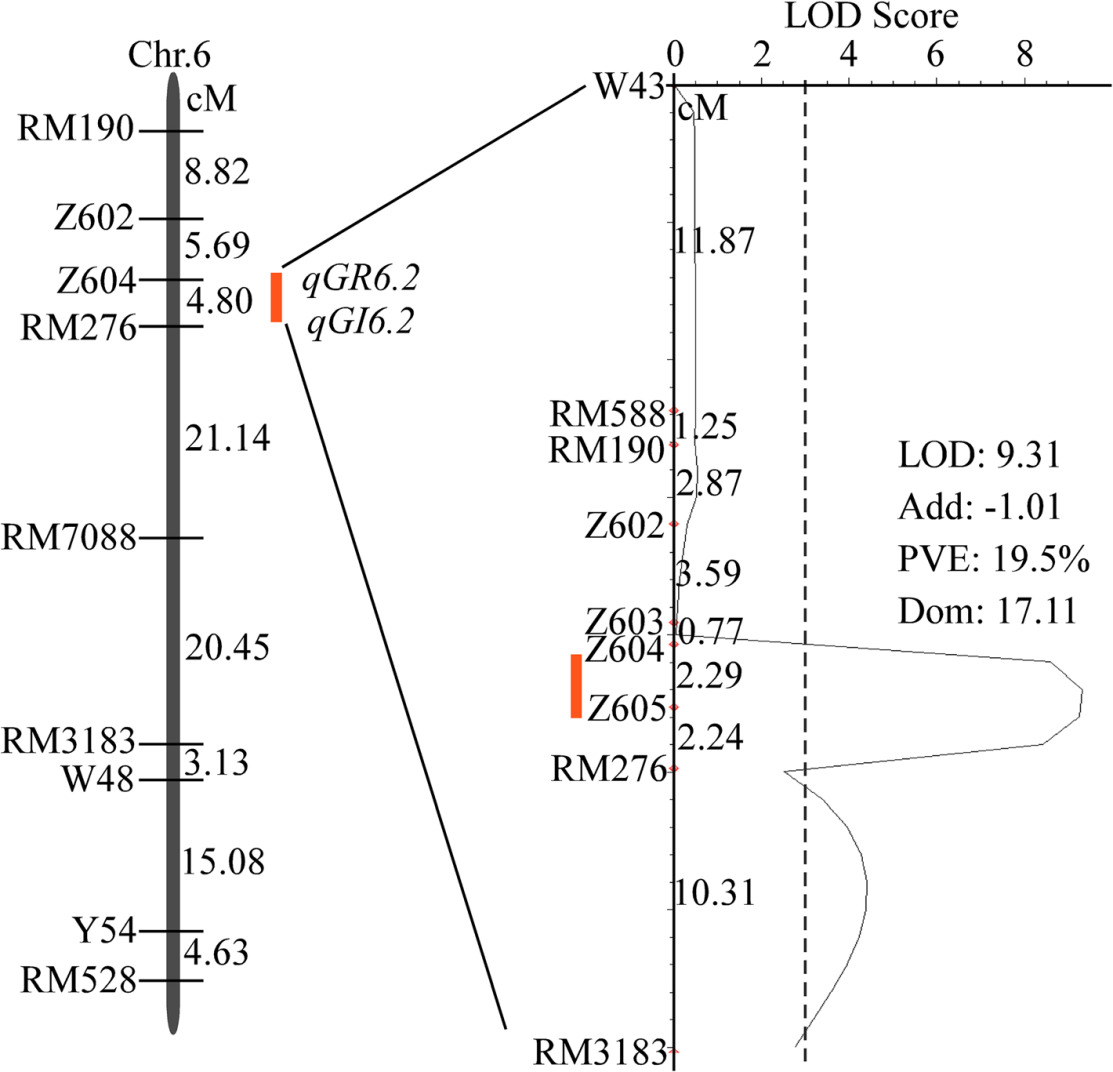
QTL mapping and validation of *qGR6.2*. On the left, the red line represents the region of QTLs among the BC_1_F_2_ population, on the right, validation of *qGR6.2* among the BC_2_F_2_ population under 300 mM NaCl conditions. The LOD curve indicates the strength of evidence for the presence of *qGR6.2* within Z604~Z605 on chromosome 6. Dashed lines show LOD thresholds of 3.0. Marker names and genetic distances are shown on the right of the chromosome. Chr., chromosome; cM, centimorgan; LOD, the likelihood of odds; PVE, phenotypic variation explained by QTL; ADD, the additive effect of substituting a WJZ allele for a Nip allele, which its negative value indicates that WJZ contains the positive allele; DOM, dominance effect

To validate the major *qGR6.2* controlling seed germination under salt stress, we further structured a BC_2_F_2_ population consisting of 70 individuals. There was a significant peak between markers Z604 and Z605 based on GR at 13 d under 300 mM NaCl conditions, and its phenotypic variation and LOD values were 19.50% and 9.31, respectively (Fig. 4). This result indicated that *qGR6.2* could improve rice seed germination under salt stress.

**Fig. 5:**
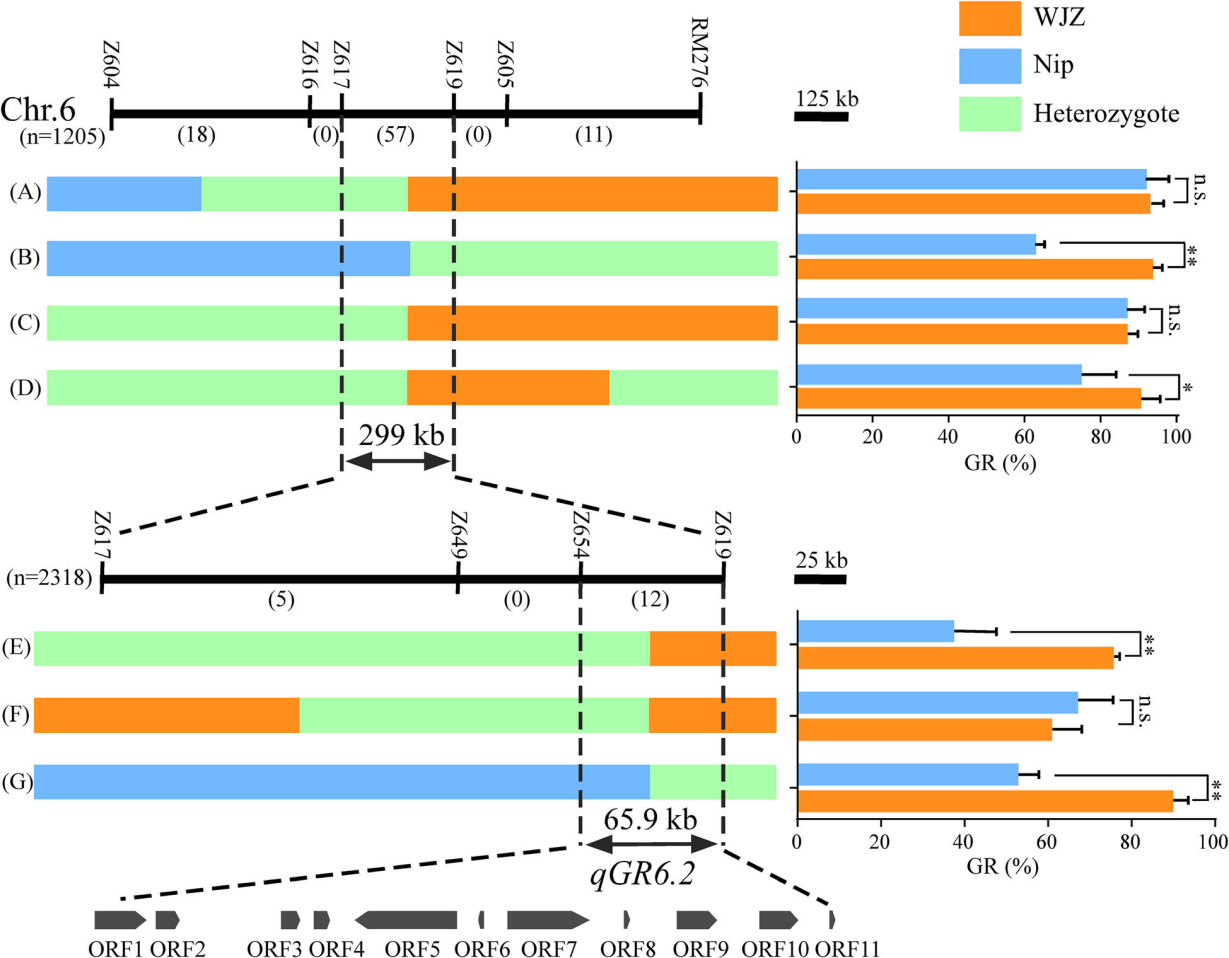
Fine mapping of *qGR6.2*. The *qGR6.2* region was narrowed down to an approximately 65.9-kb region flanked by the markers Z654 and Z619 on the short arm of chromosome 6. On the left, numbers between two markers represent recombinant individuals. A-G represents seven groups according to genotype. The physical positions of the map are based on RAP-DB (http://rapdb.lab.nig.ac.jp/index.html). On the right, the phenotype is the average GR after 10 d of seed imbibition under 300 mM NaCl conditions. All individuals for assessing GR were derived from the left corresponding recombinant progeny by selfing. Orange bars, light blue bars, and light green bars represent the WJZ, Nip, and heterozygous genotypes or corresponding phenotypes, respectively. Arrows indicate eleven ORFs in this region according to the Rice Genome Annotation Project (RGAP)

A large BC_2_F_3_ population consisting of 1205 individuals was developed to narrow the region of *qGR6.2*. Eighty-six recombinants were identified between Z604 and RM276 markers (Fig. 5). Eighteen recombinant events were between Z604 and Z616, 57 recombinant events were between Z617 and Z619, and eleven recombinant events were between Z605 and RM276 (Fig. 5). Based on the genotypes, these 86 recombinants were classified into four groups (A-D). For each group, we selected the homozygous individuals as the heterozygous region from the recombinants’ progeny by selfing. The homozygous individuals were further divided into two kinds of genotypes, one genotype is from WJZ, and the other is from Nip. Seed germination under salt stress was assessed by the average GR values of the two different genotypes. In groups B or D, the average value of GR at 10 d for homozygous WJZ alleles was significantly higher than that for Nip alleles, while there was no difference in groups A or C. *qGR6.2* was delimited between the Z617 and Z619 markers (Fig. 5). Similarly, the larger BC_2_F_4_ population derived from heterozygous BC_2_F_3_ plants in markers Z617 and Z619 was developed, containing 2318 individuals. Three types of recombination were obtained (E, F and G), consisting of 17 recombinants (Fig. 5), and the assay of each homozygous individual (BC_2_F_5_) from the recombinant group was conducted. Finally, the *qGR6.2* locus was narrowed down to a 65.9-kb region between markers Z654 and Z619 (Fig. 5).

### Prediction and expression analysis of candidate genes in the *qGR6.2* locus

According to the MSU Rice Genome Annotation Project Database (http://rice.plantbiology.msu.edu), eleven open reading frames (ORFs) were annotated within the 65.9-kb region located in the *qGR6.2* locus, including five functional proteins, one transposon protein and five expressed proteins without annotation (Table 3). Five genes with functional annotation showed that *ORF1* (*LOC_Os06g10650*) encodes a tyrosine phosphatase family protein, *ORF2* (*LOC_Os06g10660*) encodes a lysM domain-containing GPI-anchored protein 1 precursor, *ORF3* (*LOC_Os06g10670*) encodes an aspartic proteinase nepenthesin-1 precursor, *ORF5* (*LOC_Os06g10690*) encodes a PHD-finger domain-containing protein (PHD: plant homeodomain), and *ORF11* (*LOC_Os06g10750*) encodes an integral membrane protein DUF6-containing protein (DUF6: Domain of unknown function).

**Table 3:** Predicted candidate genes of *qGR6.2*

Number	Candidate genes	Putative protein function
*ORF1*	*LOC_Os06g10650*	Tyrosine phosphatase family protein, putative, expressed
*ORF2*	*LOC_Os06g10660*	LysM domain-containing GPI-anchored protein 1 precursor, putative, expressed
*ORF3*	*LOC_Os06g10670*	Aspartic proteinase nepenthesin-1 precursor, putative, expressed
*ORF4*	*LOC_Os06g10680*	Expressed protein
*ORF5*	*LOC_Os06g10690*	PHD-finger domain containing protein, putative, expressed
*ORF6*	*LOC_Os06g10700*	Expressed protein
*ORF7*	*LOC_Os06g10710*	Expressed protein
*ORF8*	*LOC_Os06g10720*	Expressed protein
*ORF9*	*LOC_Os06g10730*	Expressed protein
*ORF10*	*LOC_Os06g10740*	Transposon protein, putative, unclassified, expressed
*ORF11*	*LOC_Os06g10750*	Integral membrane protein DUF6-containing protein, expressed

**Fig. 6:**
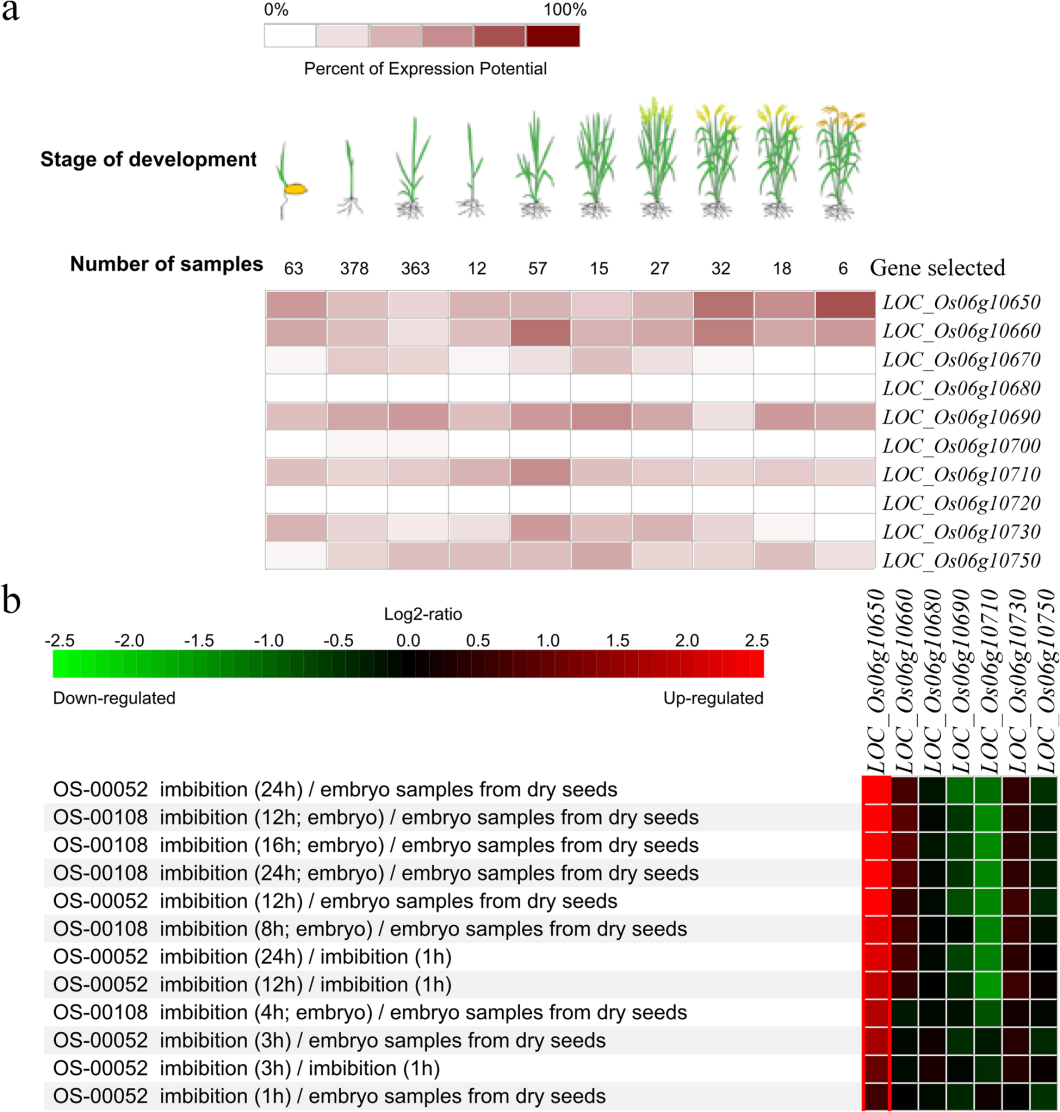
Expression patterns of candidate genes in various developmental stages (**a**) and response to seed imbibition (**b**) based on mRNA-Seq data and Affymetrix microarray datasets from GENEVESTIGATOR (http://www.genevestigator.com). The expression potential of a gene is a robust indicator for the maximal expression level of this gene. The ‘OS-nnnnn’ refers to the experiments ID

Based on RNA-Seq data and array database deposited in GENEVESTIGATOR, the expression profiles of 10 ORFs in various developmental stages and seed imbibition were obtained, except for *ORF10* encoding transposon protein (Fig. 6). The results showed that at the seed germination stage, there were higher transcript abundances for five genes, *LOC_Os06g10650*, *LOC_Os06g10660*, *LOC_Os06g10690*, *ORF7* (*LOC_Os06g10710*) and *ORF9* (*LOC_Os06g10730*), low transcript abundances for two genes, *LOC_Os06g10670* and *LOC_Os06g10750*, and almost no expression for three genes, *LOC_Os06g10680*, *LOC_Os06g10700* and *LOC_Os06g10720* (Fig. 6a). During seed germination, the expression of *LOC_Os06g10650* in the whole seeds or the isolated embryo was significantly up-regulated, and *LOC_Os06g10710* was down-regulated obviously (Fig. 6b).

**Fig. 7:**
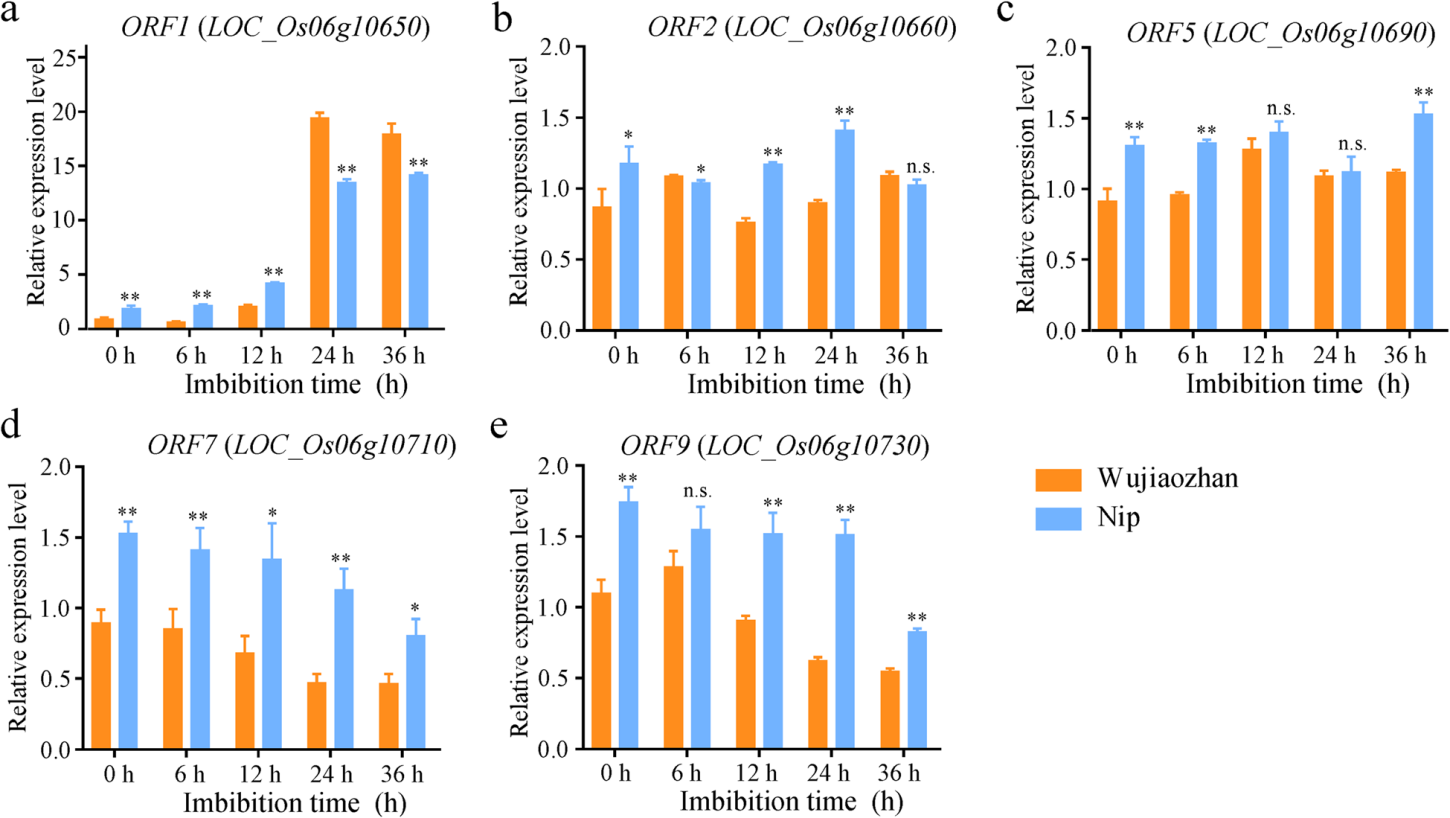
Expression levels of candidate genes between WJZ and Nip during seed germination under 300 mM NaCl conditions. Expression analysis of five ORFs (**a**-**e**) after seed imbibition for 0 h, 6 h, 12 h, 24 h and 36 h under 300 mM NaCl conditions between WJZ and Nip. Data represent the mean ± SD (*n*=3). * and ** indicate significant differences between WJZ and Nip at the 5 and 1% levels, respectively. n.s. indicates no significant difference

With the quantitative real-time PCR (RT-qPCR) approach, we subsequently detected the expression of 5 ORFs (*LOC_Os06g10650*, *LOC_Os06g10660*, *LOC_Os06g10690*, *LOC_Os06g10710* and *LOC_Os06g10730*) in WJZ and Nip during seed germination under 300 mM NaCl conditions, which showed high transcript abundances at the seed germination stage based on GENEVESTIGATOR database. The expression of *LOC_Os06g10650* was up-regulated dramatically in both parents during seed germination under 300 mM NaCl conditions (Fig. 7a). The expression of *LOC_Os06g10660* and *LOC_Os06g10690* were smooth over time (Fig. 7b, c). The expression of *LOC_Os06g10710* and *LOC_Os06g10730* was slightly down-regulated during seed germination under salt stress (Fig. 7d, e). Compared to Nip, the significant higher expression of *LOC_Os06g10650* in WJZ seeds was observed after imbibition for 24 h and 36 h. Taken together with gene function annotation and expression profiles, it indicates that *ORF1* (*LOC_Os06g10650*), encoding a tyrosine phosphatase family protein might be the causal candidate gene for seed germination under salt stress in the *qGR6.2* locus*.*

## Discussion

Salinity seriously affects rice seed germination and seedling establishment, especially in the direct-seeding area, leading to rice reduction in yields [10, 11, 20]. In this study, the *indica* landrace WJZ from Yunnan Province in China showed a strong capability of seed germination and seedling establishment under high salinity. When exposed to 300 mM NaCl, the seeds of WJZ could start to germinate after 2 days of imbibition and established normal seedlings after 5 days of imbibition. This finding suggests that WJZ is important germplasm with a strong capability of seed germination under high salinity, similar to other rice accessions N22-C-334-3 [25], Italica Livorno [23] and Jiucaiqing [24]. WJZ was considerably taller than Nip with 167.62 cm vs. 84.18 cm of plant height and fell down easily in field planting. Hence, it is of great importance to explore elite genes controlling seed germination under salt stress from WJZ, which will be beneficial for improving rice seed germination under salt stress in direct-sowing areas. As reported in previous studies, rice suffered from salinity stress during the whole growth stage, and salt tolerance at one developmental stage might not be correlated with salt tolerance at other stages [20, 26, 27]. WJZ has a very strong tolerance to salt stress at seed germination stage, however, it needs to be studied at other growth stages. Through pyramiding various salt-tolerance loci, or those loci expressing at germination, seedling, tillering or booting stages, it may be possible to develop new rice varieties with salt tolerance across all growth stages, and so improve production in rice direct-seeding areas or saline soils.

Evaluating the phenotype of salt tolerance comprehensively and accurately is the most crucial step for QTL mapping [26]. Previous studies showed that it’s a good combination of final germination rate with germination index as germination parameters [4, 28]. Both parameters showed correlation and provided reliable information on germination levels likewise temporal aspects of germination [28]. In this study, we evaluated seed germination using the parameters of GR and GI under H_2_O and NaCl conditions for the BC_1_F_2_ population that was derived from a BC_1_F_1_ individual containing approximately 37.76% genetic region of Nip (Fig. S1b). The continuous distribution and wide range of genetic variations in the BC_1_F_2_ population were found during seed germination under H_2_O and NaCl conditions, suggesting seed germination was regulated by various genes. Additionally, GR at 7 d under 300 mM NaCl conditions showed symmetrical distribution, suggesting that there was great genetic variation at this time point and might be a crucial period for breaking through the seed coat to germinate under salt stress (Fig. 2e).

In this study, a total of 13 QTLs controlling seed germination were identified via QTL mapping under H_2_O and 300 mM NaCl conditions. All these QTLs could be used to improve rice seed germination and salt tolerance through gene pyramiding by MAS in the future. By comparison, *qGR6.1*, *qGR8.1*, *qGR8.2*, *qGR10.1*, *qGR10.2* and *qGI10.1* were consistently identified under both H_2_O and 300 mM NaCl conditions, suggesting they may simultaneously regulate seed germination and salt stress. *qGR6.2*, *qGR10.1* and *qGR10.2* were identified at different time points of seed germination and shared the same region with *qGI6.2*, *qGI10.1* and *qGI10.2*, suggesting they are curial loci for seed germination under salt stress. By comparing chromosomal locations of reported QTLs, *qGR3.3*, *qGR6.2*, *qGR10.1*, *qGI6.2* and *qGI10.1* in the BC_1_F_2_ population were located in the same or adjacent regions as previously reported QTLs. *qGR3.3* was near the region of *qLTG-3-2* for low-temperature germination ability reported by Fujino et al. in 2004 [29] and *qGR3–1* for germination rate reported by Cui et al. in 2002 [30]. *qGR6.2* and *qGI6.2* were close to the *qIR-6* position for seed germination under salt stress reported by Wang et al. in 2011 [4], and one gene, *OsRR22* was located within this region and involved salt tolerance at the seedling stage reported by Takagi et al. in 2015 [31]. The regions of *qGR10.1* and *qGI10.1* were similar to *qSKC10* and *qRKC10* identified for the shoot and root potassium content under salt stress at the seedling stage [26]. These results indicated that the co-localized QTLs at the different developmental stages were the weightily genomic regions for salt tolerance in rice.

Here, we focused on the major QTL *qGR6.2*, which was associated with both GR and GI under salt conditions. At last, *qGR6.2* was mapped in a region of 65.9 kb with eleven candidate genes. Among these eleven candidate genes, only *ORF2* named *LYP6* has been reported to play dual roles in peptidoglycan and chitin perception in rice innate immunity [32]. As reported previously, PTP family proteins have been reported to regulate signal transduction and control plant growth and development [33], and the PHD finger has been identified as one of the major families of histone reader domains, being involved in recognition of methylated H3K4 [34], so we speculated *ORF1* (*LOC_Os06g10650*) and *ORF5* (*LOC_Os06g10690*) might play similar functions in rice. Based on the GENEVESTIGATOR database and RT-qPCR, the expression of *LOC_Os06g10650* was significantly up-regulated, and there was a significant higher level in WJZ than Nip during seed germination under salt conditions, suggesting important roles of *LOC_Os06g10650* in seed germination under salt stress. According to previous studies, *AtPTP1*, the first PTP family gene in the plant, was up-regulated by high salt stress [35]. Another PTP family gene *At5g23720* was reported to play import role in ABA signaling, of which mutant *phs1–3* exhibited a strong ABA-induced inhibition of seed germination in *Arabidopsis* [36]. In rice genome, there were 132-protein phosphatase-coding genes in silico investigation [37], and they were categorized into PP2A, PP2C, PTP, DSP and LMWP classes according to domain analysis and phylogenetic analysis [37, 38]. Phylogenetic relationship results revealed that *LOC_Os06g10650* (*OsPP68*) belongs to PP2C class [37]. Expression profiles showed that there were 46 genes of phosphatase family to be differentially expressed under three abiotic stress conditions (salt, cold and drought) [37]. All these imply that *LOC_Os06g10650* might be the causal candidate gene of *qGR6.2*. The function of it will be validated by genetic transformation using CRISPR/Cas9 or other method.

## Conclusions

In this study, we identified 13 QTLs for seed germination traits under H_2_O and salt conditions, which provide information on the genetic basis of improving salt tolerance during seed germination by MAS. Of these loci, the major QTL *qGR6.2*, specifically for seed germination under salt stress, was fine mapped within a region of 65.9 kb with one more likely causal gene, *LOC_Os06g10650*. Sequence analysis and genetic transformation will be carried out in the future to validate the function of the candidate gene and elucidate the molecular mechanism underlying seed germination under salt stress. The major QTL *qGR6.2* could be highly useful for improving seed germination under salt stress by the MAS strategy.

## Materials and methods

### Plant materials

The *indica* landrace WJZ from Yunnan Province in China was crossed with *japonica* Nip to generate F_1_. One F_3_ individual plant with high germinability under salt stress was selected to obtained BC_1_F_1_ seeds by backcrossing with Nip, and then a BC_1_F_1_ individual plant with high germinability under salt stress was self-crossed to generate the BC_1_F_2_ population. The BC_1_F_2_ was backcrossed with Nip to produce the BC_2_F_2_ population, and followed by self-crossed to generate BC_2_F_3_, BC_2_F_4_ and BC_2_F_5_. The BC_1_F_2_ and BC_2_F_2_ populations were used for QTLs mapping, and BC_2_F_3_, BC_2_F_4_ and BC_2_F_5_ were used for fine mapping of *qGR6.2*. All plants were grown in a paddy field at the Jiangpu Experimental Station of Nanjing Agricultural University (Jiangsu Province, China) with 17 cm between plants within a row and 33 cm between rows. The seeds of each line or individual were harvested at maturity and dried at 42 °C for 7 d to break seed dormancy and then stored at − 20 °C.

### Evaluation of seed germination under H_2_O and NaCl conditions

A total of 30 healthy grains from each line were surface-sterilized with 0.5% sodium hypochlorite solution for 15 min and then rinsed three times with sterile distilled water. Seeds were imbibed in a Petri dish (diameter 9 cm) with 40 mL quartz (diameter 1~2 mm) and 20 mL solution for 10 d under H_2_O conditions and 14 d under NaCl conditions, respectively. The different NaCl solutions (0 mM, 150 mM, 200 mM, 250 mM, 300 mM, and 350 mM) were applied for two parents to determine the fitting salt concentration of treatment. 0 mM and 300 mM NaCl solutions were used for the BC_1_F_2_ population to detect QTLs responsible for seed germination under H_2_O and NaCl conditions. The evaluation of seed germination under 300 mM NaCl was conducted for fine mapping of target QTL among the BC_2_F_2_, BC_2_F_3_, BC_2_F_4_ and BC_2_F_5_ populations. All seeds were grown at 25±1 °C in a growth chamber under 12 h light/12 h day conditions. Seed germination was defined as the emergence of the radicle (2 mm) through the surrounding tissue, and the seedling establishment was considered when the root length reached the seed length and the shoot length reached half of the seed length [21]. Germination ability was observed every day to calculate the germination rate (GR) and seedling percentage (SP). The germination index (GI) was calculated as GI = ∑ (*Gt*/t), where *Gt* is the number of germinated seeds on day *t* [4]. Three replications of each line were performed.

### DNA extraction and PCR analysis

Total genomic DNA was extracted from the young leaves of each plant using the cetyltrimethylammonium bromide (CTAB) method. PCR was performed as described by Chen et al. [39]. The PCR products were separated by electrophoresis through 8% nondenaturing polyacrylamide gels and visualized by silver staining [40].

### QTL mapping

According to the International Rice Microsatellite Initiative (IRMI, http://www.gramene.org) [41], a total of 157 SSR or InDel markers were polymorphic between WJZ and Nip and scattered on 12 chromosomes (Table S1). The BC_1_F_2_ population with 181 individuals was used to construct a genetic map by Mapmaker/Exp 3.0 [42]. GR at 2 and 3 d under H_2_O conditions and GI, GR at 5, 7, 9, 11 and 13 d and GI under 300 mM NaCl conditions were used for QTL mapping. QTL analysis was carried out by Inclusive Composite Interval Mapping (ICIM) [43] with a threshold of LOD > 3 operating 1000 permutations. The phenotypic variation and additive and dominance effects of each QTL were estimated.

### Validation and fine mapping of *qGR6.2*

Six polymorphic InDel markers were developed and used for validation and fine mapping of *qGR6.2* (Table S2). A linkage map of 70 individuals from the BC_2_F_2_ population was analyzed with 9 SSR markers (Fig. 4) on chromosome 6 to ensure the presence of the major QTL *qGR6.2*. A total of 1205 BC_2_F_3_ individuals were used to screen recombinants between the Z604 and RM276 markers, and 2318 BC_2_F_4_ individuals were used to screen recombinants between the Z617 and Z619 markers. In total, seven types of recombinants were identified. Twenty progenies of each recombinant were planted and screened for homozygous plants from each group. These homozygous plants (BC_2_F_4_, BC_2_F_5_) were tested for seed germination under 300 mM NaCl conditions. The average GR value of seed in each group after 10 d of imbibition was used for fine mapping.

### Prediction and expression analysis of candidate genes

Open reading frames (ORFs) in the region of markers Z654 and Z619 were predicted by the Rice Annotation Project Database (http://rice.plantbiology.msu.edu/). GENEVESTIGATOR (https://genevestigator.com/gv/) was employed to analyze the expression patterns of eleven candidate genes based on 2836 Affymetrix microarray datasets and 565 RNA-Seq data in seed imbibition with a significance level of *P* < 0.05.

Seeds of two parents were sampled after 0 h, 6 h, 12 h, 24 h and 36 h imbibition at 300 mM NaCl, frozen quickly in liquid nitrogen and stored at − 80 °C for RNA extraction. Total RNA was isolated from approximately 80∼100 mg powder with a total RNA Kit (BioTeke, http://www.bioteke.com). The first-strand cDNA was synthesized with random oligonucleotides using the HiScript II Reverse kit (Vazyme Biotech, http://www.vazyme.com/) according to the manufacturer’s protocol. To measure the mRNA levels of genes, RT-qPCR was conducted using a CFX96 Real-time System (Bio-Rad, USA) with SYBR Green Mix (Vazyme). The rice housekeeping gene *OsActin* (*LOC_Os03g50885*) was used as an internal control [44]. The PCR conditions were as follows: 95 °C for 5 min followed by 40 cycles of 95 °C for 15 s and 60 °C for 30 s. A final melt curve stage of 65–95 °C was performed to confirm the specificity of the primers. Relative quantification of transcript levels was obtained based on the 2^−ΔΔCT^ method [45]. The amount of the transcripts in the WJZ after imbibing for 0 h was set at 1.0. All of the primers used for RT-qPCR (Table S3) were designed according to http://quantprime.mpimp-golm.mpg.de/. Three biological replicates were conducted.

### Data analysis

The experimental data were analyzed using Statistical Analysis System (SAS) software (Cary, NC, USA) and compared with Student’s *t*-test at the 5 and 1% levels of probability.

## Supplementary Information

**Additional file 1: Figure S1.** Overview of the mapping populations and the genetic basis of BC_1_F_1_. (a) A flow chart that describes the construction of the mapping population in this study. (b) The genetic basis of a BC_1_F_1_ individual plant was derived from the backcrossing of one F_3_ single plant with Nip. The light blue, light green, and gray regions represent segments derived from Nip, heterozygous, and the centromere, on 12 chromosomes (listed as 1 to 12), respectively. The mapped markers are positioned by chromosome assignment from the high-density restriction fragment length polymorphism linkage map and described in Table S1.

**Additional file 2: Table S1.** Summary of 157 pairs of polymorphic SSR or InDel markers between WJZ and Nip.

**Additional file 3: Table S2.** Primers of PCR-based markers used for validation and fine mapping *qGR6.2*.

**Additional file 4: Table S3.** Primers for expression pattern analysis of candidate genes by real-time PCR (RT-qPCR).

## Data Availability

The WJZ seeds are provided by Dr. Luyuan Dai at Yunnan Academy of Agricultural Sciences. The datasets used and/or analysed during the current study are available from the corresponding author on reasonable request. The raw data regarding to linked genotype and phenotype for QTL mapping are available via figshare (10.6084/m9.figshare.13413674.v1).

## Abbreviations

WJZ: Wujiaozhan
Nip: Nipponbare
GR: Germination rate
SP: Seedling percentage
GI: Germination index
QTLs: Quantitative trait loci
MAS: Marker-assisted selection
GWAS: Genome-wide association studies
PVE: Phenotypic variation explained by QTL
SSR: Simple sequence repeats
InDel: Insertion/Deletion
ORF: Open reading frame
PTP: Protein tyrosine phosphatase
CTAB: Cetyltrimethylammonium bromide
RT-qPCR: Quantitative real-time PCR
