# Supplementary figures and images for: Identification and fine mapping of qGR6.2, a novel locus controlling rice seed germination under salt stress
Source: BMC Plant Biol. 2021 Jan 9;21:36. doi: 10.1186/s12870-020-02820-7 (PMC7797128; doi:10.1186/s12870-020-02820-7)

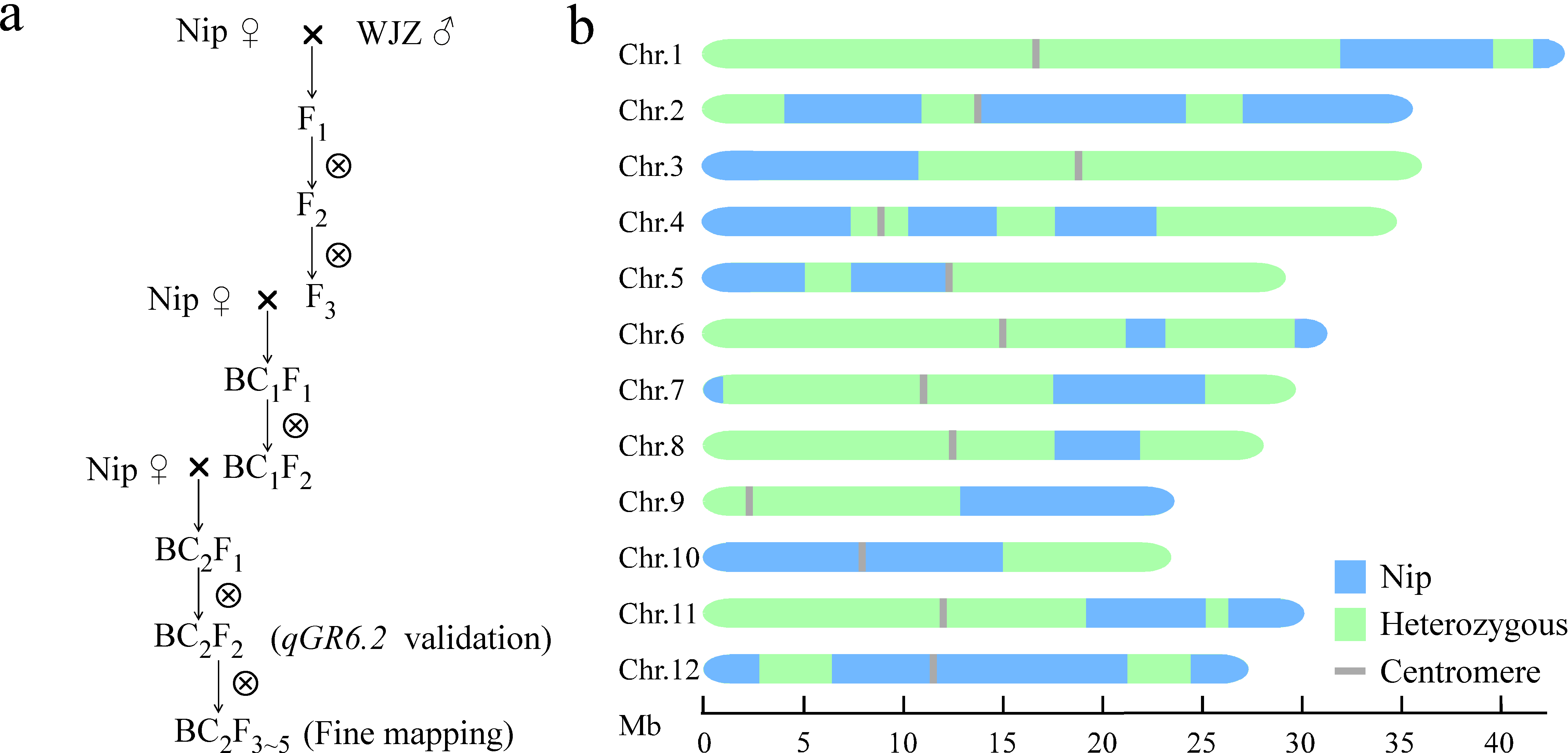

Supplement: Supplementary file 1 — Additional file 1: Figure S1. Overview of the mapping populations and the genetic basis of BC1F1. (a) A flow chart that describes the construction of the mapping population in this study. (b) The genetic basis of a BC1F1 individual plant was derived from the backcrossing of one F3 single plant with Nip. The light blue, light green, and gray regions represent segments derived from Nip, heterozygous, and the centromere, on 12 chromosomes (listed as 1 to 12), respectively. The mapped markers are positioned by chromosome assignment from the high-density restriction fragment length polymorphism linkage map and described in Table S1. [file 12870_2020_2820_MOESM1_ESM.tif]
